# B-type natriuretic peptide ability to predict mortality after transcatheter aortic valve replacement

**DOI:** 10.2459/JCM.0000000000001230

**Published:** 2021-08-23

**Authors:** Heidi Lehtola, Jarkko Piuhola, Matti Niemelä, Tuomas Tauriainen, Juhani Junttila, Timo Mäkikallio, Tatu Juvonen, Fausto Biancari

**Affiliations:** a Department of Internal Medicine, Oulu University Hospital, Oulu; b Heart and Lung Center, Helsinki University Hospital and University of Helsinki; c University of Helsinki, Helsinki; d Research Unit of Surgery, Anesthesia and Critical Care, Faculty of Medicine, University of Oulu, Oulu, Finland

**Keywords:** B-type natriuretic peptide, transcatheter aortic valve implantation, transcatheter aortic valve replacement

## To the Editor

An increased baseline brain natriuretic peptide (BNP)/pro-BNP level is predictive of mortality after transcatheter aortic valve replacement (TAVR) for aortic stenosis.^1–9^ In this study, we investigated whether BNP may have an incremental prognostic effect in these patients.

## Methods

This study includes data on 293 patients who underwent TAVR with newer generation devices [Evolut R (Medtronic Inc, Minneapolis, Minnesota, USA) *N* = 50; Sapien 3 (Edwards Lifesciences, Irvine, California, USA) *N* = 190; ACURATE neo (Boston Scientific, Marlborough, Massachusetts, USA) *N* = 36; Lotus (Boston Scientific, Marlborough, Massachusetts, USA) *N* = 17] at the Oulu University Hospital, Finland, from 2014 to 2017. The Institutional Review Board approved this study. Mortality data were obtained from the Population Register Center with a follow-up coverage of 100%.

Statistical analysis was performed with Stata v. 15.1 software (StataCorp LLC, Texas, USA). The area under the curve (AUC) of the receiver-operating characteristics (ROC) curve was estimated to assess the discrimination ability of the baseline BNP to predict mortality. The Youden's test was used to identify the best cutoff of BNP in predicting outcomes. Logistic regression and the Cox proportional hazard method were used for multivariate analysis. The DeLong test was used to assess the difference between the AUCs of different regression models. *P* less than 0.05 was set for statistical significance.

## Results

**Fig. 1:**
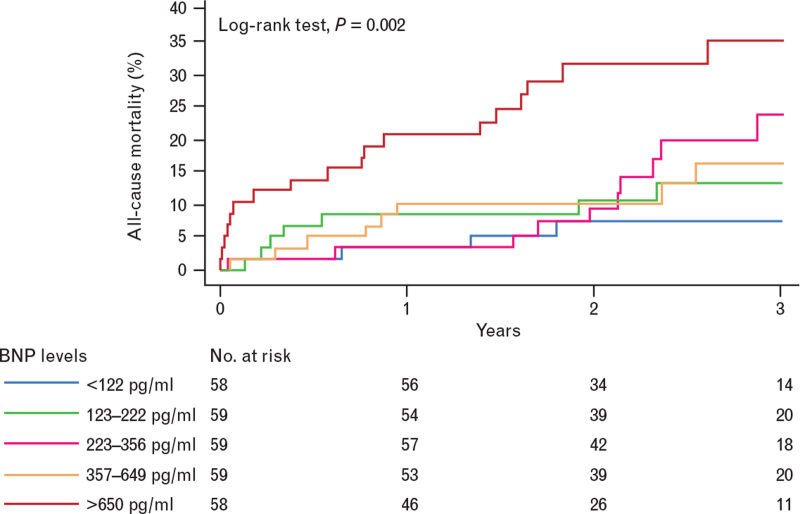
Survival according to baseline brain natriuretic peptide quintiles (log-rank test, *P* < 0.0001).

The data of the patient cohort are summarized in Table 1. Transfemoral access was used in 276 (94.2%) patients. Mean follow-up was 2.3 ± 1.0 years. Mortality was 3.1% at 30 days and 19.4% at 3 years. Late mortality increased along with increasing quintiles of baseline BNP (Fig. 1).

**Table 1:** Baseline characteristics of patients undergoing transcatheter aortic valve replacement and risk estimates of 30-day and 3-year mortality

		30-day mortality	Three-year mortality
	No. (%)/mean (SD)	OR, 95%CI	(HR, 95% CI)
Age (years)	82.1 (6.1)		
Female	158 (53.9)		
Brain natriuretic peptide (pg/ml)	471 (578)	^a^3.241, 1.431–7.343	^a^1.527, 1.105–2.109
Anemia	105 (35.8)		1.912, 1.026–3.561
eGFR (ml/min/1.73 m^2^)	63 (18.0)		0.984, 0.969–0.999
Diabetes	89 (30.4)		
Coronary artery disease	104 (35.5)	10.015, 1.688–59.440	
Recent myocardial infarction	2 (0.7)		
Prior cardiac surgery	47 (16.0)		
Prior percutaneous coronary intervention	69 (23.5)		
Critical preoperative state/acute heart failure	27 (9.2)		
Prior aortic balloon valvuloplasty	13 (4.4)		
Prior stroke	28 (9.6)		
Extracardiac arteriopathy	57 (19.5)	5.895, 1.228–28.308	
Atrial fibrillation	117 (39.9)		
Prior pacemaker	29 (9.9)		
Pulmonary disease	76 (25.9)		
NYHA functional classes			
I	2 (0.7)		
II	47 (16.0)		
III	233 (79.5)		
IV	11 (3.8)		
LVEF ≤50%	68 (23.2)		
Moderate/severe mitral valve regurgitation	58 (20.2)		
SPAP			
31–55 mmHg	136 (46.4)		
>55 mmHg	41 (14.0)		
EuroSCORE II (%)	7.1 (8.3)		
STS score (%)	4.0 (2.3)		

Continuous variables are reported as means ± standard deviation (SD) and categorical variables as counts and percentages. CI, confidence interval; eGFR, glomerular filtration estimated according to the CKD-EPI equation; HR, hazard ratio; LVEF, left ventricular ejection fraction; NYHA, New York Heart Association; OR, odds ratio; PCI, percutaneous coronary intervention; SPAP, systemic pulmonary artery pressure; STS, Society of Thoracic Surgeons.

a Logarithmically transformed; clinical variables are according to the EuroSCORE II definition criteria.

When baseline BNP was transformed into its natural logarithm, it was an independent predictor of 30-day [odds ratio (OR) 2.241, 95% confidence interval (CI) 1.431–7.343] and 3-year mortality (hazard ratio 1.527, 95% CI 1.105–2.209) (Table 1). The ROC AUC for 30-day mortality was 0.752 (95%CI 0.572–0.932) and for 3-year mortality was 0.657 (95% CI 0.573–0.741). The best cutoff of baseline BNP was 690 pg/ml for 30-day mortality (11.3 vs. 1.3%, adjusted OR 11.936, 95% CI 2.369–60.144), and it had an impact also on 3-year mortality (36.1 vs. 15.8%, adjusted hazard ratio 2.451, 95% CI 1.299–4.623).

Logistic regression showed that baseline BNP either as a continuous variable or as cutoff of baseline BNP of 690 pg/ml was not predictive of 30-day mortality when adjusted for EuroSCORE II and STS score. Cox proportional hazard analysis showed that baseline BNP as a continuous variable was not predictive of 3-year mortality when adjusted for EuroSCORE II and STS score. However, a cutoff of baseline BNP was 690 pg/ml was predictive of 3-year mortality either adjusted for EuroSCORE II (hazard ratio 2.110, 95%CI 1.085–4.105) or STS score (hazard ratio 1.936, 95% CI 1.005–3.729).

## Comment

The present study confirmed that BNP was an independent predictor of early and late mortality after TAVR when adjusted for other significant variables.^1–8^ A few studies investigated whether BNP/pro-BNP may increase the predictive ability of current risk-scoring methods in patients undergoing cardiovascular interventions but not all studies were able to demonstrate this effect when BNP/pro-BNP was added to the EuroSCORE or STS scores.^10–14^ In the present study, baseline BNP levels were predictive of 30-day and 3-year mortality when adjusted for other independent clinical risk factors. However, when adjusted for the STS score and the EuroSCORE II, only BNP dichotomized according to its cutoff value of 690 pg/ml was predictive of 3-year mortality. We speculate that the relatively small size of this series might lead to type II error.

## Conclusion

The present findings showed that baseline BNP levels were predictive of early and mid-term mortality after TAVR with newer generation devices. However, the incremental prognostic value of BNP in TAVR over current risk scores may be limited and should be further assessed in larger studies.

### Conflicts of interest

There are no conflicts of interest.
